# Pre-Type 1 Diabetes in Adolescents and Teens: Screening, Nutritional Interventions, Beta-Cell Preservation, and Psychosocial Impacts

**DOI:** 10.3390/jcm14020383

**Published:** 2025-01-09

**Authors:** Brody Sundheim, Krish Hirani, Mateo Blaschke, Joana R. N. Lemos, Rahul Mittal

**Affiliations:** 1 Young Leaders Advocacy Group, Diabetes Research Institute Foundation, Hollywood, FL 33021, USA; brosundheim@gmail.com (B.S.); krish.ada@outlook.com (K.H.); mblaschke10@gmail.com (M.B.); joanalemos@miami.edu (J.R.N.L.); 2 Ransom Everglades High School, 3575 Main Hwy, Miami, FL 33133, USA; 3 Diabetes Research Institute, University of Miami Miller School of Medicine, Miami, FL 33136, USA; 4 American Heritage School, 12200 W Broward Blvd, Plantation, FL 33325, USA; 5 Coral Gables High School, 450 Bird Rd, Coral Gables, FL 33146, USA

**Keywords:** type 1 diabetes, youth screening, nutritional intervention, family support, beta-cell preservation

## Abstract

Type 1 Diabetes (T1D) is a progressive autoimmune disease often identified in childhood or adolescence, with early stages detectable through pre-diabetic markers such as autoantibodies and subclinical beta-cell dysfunction. The identification of the pre-T1D stage is critical for preventing complications, such as diabetic ketoacidosis, and for enabling timely interventions that may alter disease progression. This review examines the multifaceted approach to managing T1D risk in adolescents and teens, emphasizing early detection, nutritional interventions, beta-cell preservation strategies, and psychosocial support. Screening for T1D-associated autoantibodies offers predictive insight into disease risk, particularly when combined with education and family resources that promote lifestyle adjustments. Although nutritional interventions alone are not capable of preventing T1D, certain lifestyle interventions, such as weight management and specific nutritional choices, have shown the potential to preserve insulin sensitivity, reduce inflammation, and mitigate metabolic strain. Pharmacological strategies, including immune-modulating drugs like teplizumab, alongside emerging regenerative and cell-based therapies, offer the potential to delay disease onset by protecting beta-cell function. The social and psychological impacts of a T1D risk diagnosis are also significant, affecting adolescents’ quality of life, family dynamics, and mental health. Supportive interventions, including counseling, cognitive-behavioral therapy (CBT), and group support, are recommended for managing the emotional burden of pre-diabetes. Future directions call for integrating universal or targeted screening programs within schools or primary care, advancing research into nutrition and psychosocial support, and promoting policies that enhance access to preventive resources. Advocacy for the insurance coverage of screening, nutritional counseling, and mental health services is also crucial to support families in managing T1D risk. By addressing these areas, healthcare systems can promote early intervention, improve beta-cell preservation, and support the overall well-being of adolescents at risk of T1D.

## 1. Introduction

Type 1 Diabetes (T1D) is a chronic autoimmune disorder primarily characterized by the destruction of insulin-producing beta cells in the pancreas, leading to an absolute deficiency in insulin production [1,2,3,4]. While T1D often manifests in childhood or adolescence, the natural history of the disease indicates that beta-cell destruction and autoimmune activation may begin years before the clinical onset [5,6]. Adolescents and teens with T1D experience lifelong challenges, including metabolic instability, the risk of severe acute complications, and an increased likelihood of developing secondary complications over time [7,8,9,10,11]. As such, understanding the early stages of T1D development, particularly the transition from pre-diabetes to overt T1D has become critical in recent years to identify effective preventive strategies.

### 1.1. Overview of Type 1 Diabetes in Adolescents and Teens

T1D is the predominant form of diabetes in young populations, with peak incidence occurring between the ages of 10 and 14 [12,13]. Genetic susceptibility, environmental triggers, and immune dysregulation are all implicated in the pathogenesis of T1D, contributing to the progressive loss of beta cells [14,15,16]. In adolescents and teens, this disease not only disrupts metabolic homeostasis but also imposes significant psychosocial burdens as children navigate a chronic, life-altering condition during their formative years. This disease’s pathophysiology in youth is often more aggressive than in adults, with faster rates of beta-cell decline, a more challenging insulin management profile, and an increased risk of diabetic ketoacidosis (DKA) at diagnosis [17,18]. The high incidence of T1D in adolescents highlights the need for strategies aimed at early detection and intervention in this vulnerable age group.

### 1.2. Pre-Diabetes as an Intermediary Stage Characterized by Insulin Resistance, Autoantibody Presence, and Subclinical Beta-Cell Dysfunction

Pre-diabetes in the context of T1D, often referred to as the “stages of T1D”, is defined by the presence of diabetes-associated autoantibodies without overt hyperglycemia [17,18,19]. Unlike pre-diabetes associated with Type 2 Diabetes, which is primarily characterized by insulin resistance, pre-diabetes in T1D is marked by an autoimmune-mediated attack on beta cells [1,20]. This phase can be stratified into stages based on the presence and progression of specific autoantibodies (such as GAD65, IA-2, and ZnT8) and glycemic abnormalities that reflect subclinical beta-cell dysfunction [17,19,21,22,23] (Table 1).

**Figure 1:**
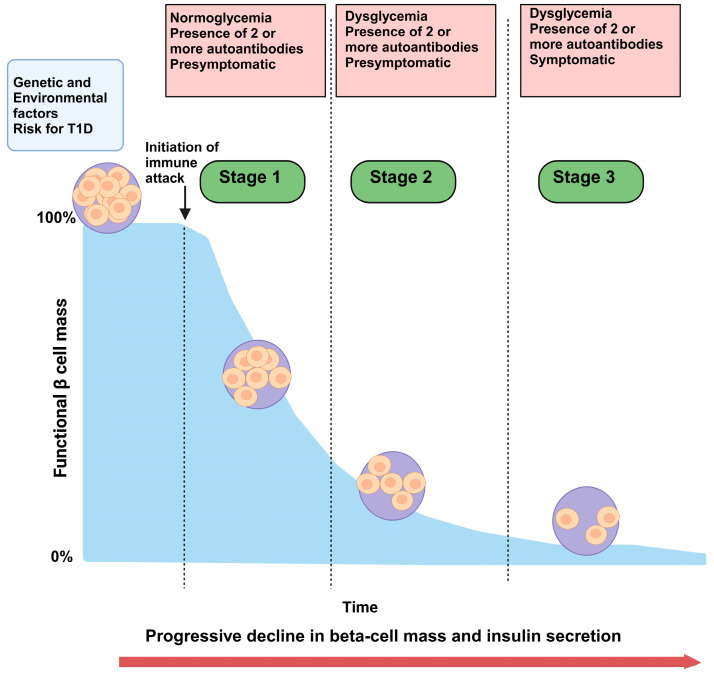
Progressive stages of beta-cell dysfunction in Type 1 Diabetes (T1D). The figure illustrates the gradual decline in functional beta-cell mass over time, highlighting the progression through three stages of T1D development. Stage 1 represents the initiation of the immune attack in individuals with normoglycemia and the presence of two or more autoantibodies, indicating a presymptomatic phase. In Stage 2, dysglycemia occurs, with the continued presence of two or more autoantibodies, yet this is still a presymptomatic state. By Stage 3, dysglycemia persists with symptoms, as beta-cell mass and insulin production are severely compromised. The figure highlights the impact of genetic and environmental factors contributing to T1D risk, leading to a progressive decline in beta-cell mass and insulin secretion. Created with Biorender.com.

Early-stage pre-diabetes (Stage 1) is identified by multiple islet autoantibodies without dysglycemia, whereas Stage 2 is marked by the onset of dysglycemia alongside persistent autoimmunity (Figure 1). In Stage 3, diagnostic criteria for diabetes are met, typically when significant beta-cell loss has occurred (Figure 1). This staging framework allows for a progressive understanding of T1D development, where each stage represents a distinct shift in the autoimmune and metabolic processes leading to clinical T1D [19]. The identification of autoantibodies at these early stages provides a predictive tool for assessing T1D risk, especially in individuals with a family history of the disease or other genetic predispositions.

Beta-cell dysfunction in the pre-diabetic stage is both autoimmune-driven and mediated by inflammatory processes. Mechanisms such as cytokine release and immune cell infiltration contribute to beta-cell apoptosis, while metabolic stress from immune-mediated inflammation further impairs insulin production. The measurement of C-peptide levels and oral glucose tolerance tests can be used to assess beta-cell function [29,30,31,32,33,34,35] (Table 1). Recent studies in adolescents and children have utilized continuous glucose monitoring (CGM) data to detect early hyperglycemia and assess its potential as a diagnostic tool for T1D. By monitoring the percentage of time participants spend above 140 mg/dL, CGM helps characterize individuals who are progressing to Stage 3 T1D [36,37,38,39,40,41,42,43] (Table 1). CGM can be particularly valuable in monitoring individuals identified as being at high risk for T1D, such as those with a family history or the presence of diabetes-related autoantibodies [17,18,19]. Research has shown that early markers of dysglycemia, including rising fasting glucose, increased postprandial glucose excursions, and greater glycemic variability, often occur during the pre-symptomatic stages of T1D, even before clinical diagnosis [41,43,44,45]. CGM can detect these subtle abnormalities, providing an opportunity for closer monitoring and potential early interventions to delay disease onset or mitigate complications. Moreover, CGM data can identify patterns of glycemic dysregulation in high-risk individuals before the onset of overt hyperglycemia [46]. Such insights highlight the utility of CGM as a monitoring tool in targeted populations, even if it may not be applicable to the general population.

The early-stage period is critical for intervention, as targeted therapies aimed at preserving beta-cell function or modulating the immune response could potentially delay or prevent the onset of clinical T1D.

### 1.3. Importance of Early Identification and Intervention, Particularly in High-Risk Populations

The early identification of pre-diabetes in adolescents and teens is a pivotal component of diabetes prevention strategies [17]. Screening for islet autoantibodies in high-risk populations, such as those with a family history of T1D or specific genetic markers (e.g., HLA-DR3/DR4 alleles), enables the early stratification of individuals based on their likelihood of disease progression [19,21,47,48]. Moreover, early identification provides a window of opportunity for intervention during the pre-diabetes phase, where immunomodulatory therapies may alter the disease trajectory [17].

Interventional studies in at-risk youth have shown promise in modulating immune responses and preserving residual beta-cell function. For example, therapies that target specific immune pathways, such as T-cell modulation, are increasingly recognized in clinical practice. Teplizumab, an FDA-approved anti-CD3 monoclonal antibody, has already been established as an infusion therapy to delay the onset of T1D [49,50,51,52]. Trials with teplizumab have reported reductions in the overall autoimmune response [53]. Additionally, emerging strategies such as regulatory T-cell preservation remain under investigation for their potential to complement and enhance the effectiveness of existing treatments [54].

The dynamics of T1D-related autoantibodies reflect a complex interplay of natural immune processes, disease progression, and potential therapeutic interventions [55]. While persistent autoantibody positivity remains a hallmark of T1D pathogenesis, transient or declining autoantibody levels have been observed in specific contexts, such as low-risk populations or in response to immunotherapy [55]. These findings emphasize the need for longitudinal monitoring and standardized methodologies to enhance our understanding of autoantibody behavior and its implications for T1D prediction and treatment.

In addition to promising recent pharmacological strategies aimed at delaying T1D progression, psychosocial support for adolescents identified as high-risk is recommended [56]. The emotional and psychological burden associated with the risk of developing a chronic illness cannot be overstated, particularly in a population already facing the challenges of adolescence. Structured education programs, counseling, and community support can help these individuals and their families cope with the stress of T1D risk and foster proactive engagement in preventive health practices [56].

This review examines pre-diabetes in adolescents and teens at risk for T1D, synthesizing current knowledge and addressing significant gaps in the literature. By highlighting the intersection of screening advancements, nutritional interventions, beta-cell preservation strategies, and psychosocial impacts, this review brings a multidisciplinary perspective that is essential for holistic T1D prevention and management. It aims to provide clinicians, researchers, and policymakers with actionable insights into the preclinical stages of T1D, emphasizing the importance of early detection and integrated care approaches.

## 2. Screening and Early Detection of Autoantibodies in Adolescents and Teens

The early detection of pre-diabetes in youth populations at risk of progressing to T1D has become a focal point in early treatment research. Screening for autoantibodies, which signal an autoimmune response against insulin-producing beta cells, can identify at-risk adolescents before they exhibit clinical symptoms, enabling the use of strategies to delay disease onset and reduce the risk of DKA [57]. This section discusses the importance and methodologies of autoantibody screening, recent advances in screening tools, and the ethical considerations that accompany early T1D risk detection.

### 2.1. Prevalence and Importance of Screening

#### 2.1.1. Rising Prevalence of Pre-Diabetes and T1D Risk in Youth Populations

In recent years, the prevalence of pre-diabetes and T1D risk factors has shown a marked increase among adolescents and teens, attributed to both genetic and environmental factors. Unlike Type 2 Diabetes, T1D is less associated with lifestyle risk factors, yet recent studies highlight a complex interplay between genetic predisposition, immune dysregulation, and environmental triggers (e.g., viral infections and dietary factors) that accelerate T1D risk even in previously unaffected youth [14,15,16]. Current estimates indicate that approximately 0.3% of adolescents in the U.S. are at high risk for T1D, evidenced by the presence of multiple islet autoantibodies [58]. It is important to note that T1D differs from Maturity-Onset Diabetes of the Young (MODY) in its underlying pathophysiology and genetic basis [59]. While T1D is an autoimmune disease characterized by the destruction of insulin-producing beta cells, MODY is a monogenic form of diabetes caused by mutations in a single gene affecting beta-cell function [60]. Despite the strong genetic predisposition in T1D, it typically involves polygenic inheritance and environmental triggers, unlike MODY, which follows an autosomal dominant inheritance pattern and lacks an autoimmune component [61].

#### 2.1.2. Utility of Autoantibody Screening in Identifying At-Risk Individuals

The utility of autoantibody screening in early detection is well-established, as the presence of diabetes-associated autoantibodies significantly increases the likelihood of developing T1D [19,21,62] (Table 1). Key autoantibodies, including those against glutamic acid decarboxylase (GAD65), insulin (IAA), islet antigen-2 (IA-2), and zinc transporter 8 (ZnT8), serve as biomarkers for beta-cell autoimmunity [19,21,62]. The presence of one autoantibody may indicate risk, but the detection of multiple autoantibodies correlates with a nearly inevitable progression to T1D, particularly in youth populations with a family history of the disease. Autoantibody screening not only identifies high-risk individuals but also aids in staging T1D progression and timing intervention strategies to preserve beta-cell function before a significant loss occurs.

### 2.2. Screening Strategies and Recommendations

#### 2.2.1. Current Guidelines and Recommendations for Screening

Leading diabetes organizations, including the Breakthrough T1D (Formerly Juvenile Diabetes Research Foundation; JDRF) and the American Diabetes Association (ADA), have established guidelines for autoantibody screening in high-risk pediatric populations [63]. These guidelines recommend screening for children with a first-degree relative with T1D, particularly between the ages of 2 and 18, as this is the period of highest risk. Screening is also encouraged for youth with specific genetic markers associated with T1D susceptibility, such as HLA-DR3 and HLA-DR4 alleles [16,24,25,26,27,28] (Table 1). Guidelines suggest a stepwise approach, where a positive initial autoantibody screening is followed by regular monitoring to assess disease progression and allow for timely intervention [64,65].

#### 2.2.2. Community Screening Programs

In recent years, community screening programs, such as the TrialNet Pathway to Prevention and The Environmental Determinants of Diabetes in the Young (TEDDY) study, have facilitated widespread screening and contributed significantly to our understanding of T1D progression [57]. These programs employ standardized methodologies for autoantibody detection and follow at-risk youth longitudinally, enabling early detection and intervention on a broad scale. Data from these programs have highlighted the potential of community-wide screening in reducing T1D-associated complications at diagnosis and identifying intervention points to delay disease onset [57].

### 2.3. Advances in Screening Tools

#### 2.3.1. Emerging Biomarkers Beyond Autoantibodies

While autoantibody screening remains the cornerstone of T1D risk detection, recent advances have identified additional biomarkers that enhance screening accuracy. Metabolomic profiling, which assesses patterns of metabolites in blood, has shown promise in distinguishing at-risk youth from those unlikely to progress to T1D. Studies indicate that changes in lipid metabolites, amino acid levels, and short-chain fatty acids are associated with T1D onset [66,67,68,69], suggesting that metabolomic markers could provide additional predictive value.

Genomic biomarkers, including specific gene variants related to immune regulation and beta-cell function, also offer the potential to refine risk assessment [17]. For example, polymorphisms in genes associated with cytokine production and immune cell signaling (e.g., IL-2, CTLA-4) have been linked to T1D susceptibility and progression [70,71]. However, these biomarkers are still in the early stages of development, and a considerable path lies ahead before they can be fully utilized in clinical settings. Integrating these genetic markers with autoantibody data may enable a more individualized risk prediction model, which could ultimately guide personalized intervention strategies.

#### 2.3.2. Evaluating Sensitivity, Specificity, and Predictive Value of Novel Biomarkers

The effectiveness of emerging biomarkers relies on their sensitivity, specificity, and predictive value, especially in diverse populations. Current studies aim to validate these markers in different ethnic and genetic backgrounds to ensure reliability. The inclusion of metabolomic and genomic markers alongside autoantibodies has been shown to increase predictive accuracy, with specificity rates nearing 90% in some cohorts [72,73,74,75,76]. The continuous improvement of these markers will be essential to refining risk prediction models and expanding screening applicability to general populations beyond those with a family history of T1D.

### 2.4. Ethical Considerations

#### 2.4.1. Ethical Implications of Screening for T1D in Light of Emerging Therapeutic Interventions

Screening asymptomatic youth for T1D risk now carries new ethical considerations, especially with the recent availability of FDA-approved therapies, such as teplizumab, that may delay disease progression [49,50,51,52]. Identifying autoantibodies and predicting T1D onset can empower families with options for early intervention, potentially altering the disease course [17]. However, the psychological impact of a preclinical diagnosis, which may include anxiety or stress over long-term outcomes, remains a concern [77]. Families must balance the promise of emerging therapies with the emotional and logistical implications of engaging in early intervention strategies. Providing targeted counseling and support is essential to help families make informed decisions about risk-based treatment and to address the complex psychosocial aspects of a preclinical T1D diagnosis [77].

#### 2.4.2. Family Counseling and Implications for Early Lifestyle Adjustments

Informed consent, education, and counseling are essential components of the T1D screening process [64]. Families should be fully informed of the implications of screening results, including the limitations of predictive tools and the possibility of false positives or uncertainties in progression [57]. Moreover, lifestyle recommendations following a positive screening result must be carefully managed to avoid undue burden on youth and their families [78]. Nutritional and behavioral interventions should focus on promoting general health benefits without fostering restrictive or unsustainable lifestyle changes [78]. Family-centered counseling can provide support and empower families to adopt manageable, health-promoting adjustments that may reduce metabolic stress on beta cells without significantly altering their quality of life [79].

## 3. Role of Nutrition in Pre-Diabetes Progression

### 3.1. Nutritional Interventions and Lifestyle Modification

Nutritional interventions and lifestyle modifications can help in managing pre-diabetes in adolescents and teens at elevated risk for T1D (Table 2) [80,81]. Although nutritional and lifestyle modifications cannot prevent T1D onset, they can enhance metabolic resilience, reduce complications, and improve the quality of life for individuals predisposed to the disease.

Current studies suggest that a balanced, nutrient-dense diet stabilizes blood glucose levels, promotes insulin sensitivity, and reduces inflammation, which is an essential factor for protecting beta cells from stress [86,87]. Low-glycemic and high-fiber diets, combined with a focus on healthy fats and proteins, can improve glucose regulation and inflammatory response modulation [88,89]. Additionally, micronutrients such as Vitamin D and Omega-3 fatty acids have been hypothesized to have the potential for immune support and beta-cell preservation, though further research is warranted to clarify their role in disease progression [90,91,92,93,94,95].

Physical activity, encompassing aerobic and resistance training, consistently benefits insulin responsiveness, cardiovascular health, and inflammation reduction. Even moderate exercise, such as daily walking, significantly supports both metabolic and mental health, providing a holistic approach to managing pre-diabetes [78]. Physical activity is further linked to improved mental well-being, helping to reduce anxiety and improve mood, which is a critical aspect for adolescents facing T1D risk [96].

It has been suggested that lifestyle interventions in T1D offer more than metabolic advantages (Table 2); they provide a proactive, empowering approach that fosters confidence, resilience, and a positive stance toward health management [78]. Structured support through counseling and educational initiatives can help families make informed, sustainable choices, mitigating the psychosocial stress often associated with a high-risk T1D status [97].

Hence, while lifestyle and nutritional modifications do not prevent T1D onset, they equip individuals and families with effective, practical strategies that positively impact health trajectories [78]. By prioritizing education, resilience, and metabolic optimization, these interventions set a strong foundation for improved outcomes and ease of transition should T1D develop [78].

#### 3.1.1. Impact of Dietary Patterns on Insulin Resistance and Inflammation

Dietary patterns profoundly influence insulin sensitivity and inflammatory pathways, shaping the progression of pre-diabetes and health outcomes for those at risk for T1D. Diets high in sugars and saturated fats are associated with increased insulin resistance and a pro-inflammatory state, exacerbating beta-cell stress and accelerating beta-cell loss [98,99,100]. Adolescents consuming diets rich in sugary beverages, processed foods, and high-fat snacks face a heightened risk of metabolic imbalances that contribute to hyperglycemia, oxidative stress, and chronic inflammation [61]. This detrimental cycle—characterized by decreased insulin sensitivity, overcompensation by beta cells, and advancing autoimmune attacks—highlights the need for dietary interventions to mitigate these effects.

#### 3.1.2. Evidence-Based Dietary Guidelines

To counteract these risks, evidence-based dietary guidelines advocate for low glycemic index (GI) diets, plant-based foods, and anti-inflammatory nutrition [78]. A low GI diet featuring whole grains, legumes, and fiber-rich vegetables prevents rapid blood sugar spikes and reduces insulin demand, alleviating metabolic strain on beta cells [61]. Meta-analyses confirm that low GI diets improve glucose regulation and support beta-cell function [80].

Plant-based diets emphasizing fruits, vegetables, legumes, and whole grains are linked to lower inflammatory markers and improved insulin sensitivity, potentially delaying T1D progression [62]. The benefits of such diets include reduced oxidative stress and improved immune function, further supporting their role in pre-diabetes management [101].

Anti-inflammatory foods, rich in Omega-3 fatty acids, antioxidants, and phytonutrients (such as leafy greens, berries, and nuts), are effective in reducing systemic inflammation—a major contributor to autoimmune activity. Emerging evidence highlights the potential of these nutrients in modulating immune responses and preserving beta-cell health [78].

### 3.2. Specific Nutrients and Dietary Supplements

#### 3.2.1. Role of Specific Nutrients in Insulin Sensitivity and Inflammation Reduction

Certain nutrients have been studied for their roles in promoting insulin sensitivity and reducing inflammation, both of which are vital for at-risk youth. Omega-3 fatty acids from fish and flaxseed oils are known to modulate inflammatory pathways and improve cell membrane integrity, thereby enhancing insulin sensitivity [102]. Antioxidants, such as vitamins C and E, neutralize free radicals and decrease oxidative stress on beta cells, supporting overall cell health [103].

Dietary fiber, particularly soluble fiber from sources like oats, legumes, and certain fruits, aids in regulating blood glucose by slowing carbohydrate absorption and improving insulin sensitivity. Fiber also supports gut microbiome health, which may indirectly influence inflammation and immune responses, adding a potential benefit for individuals at risk of autoimmune conditions like T1D.

#### 3.2.2. Potential Benefits of Vitamin D, Zinc, and Magnesium

Vitamin D plays a dual role in immune function and insulin sensitivity, and deficiency in vitamin D has been linked to a higher risk of autoimmune diseases, including T1D [104,105,106]. Supplementation of this vitamin in at-risk youth may help to modulate immune responses and preserve beta-cell function [107,108,109,110]. Magnesium, an essential mineral involved in glucose metabolism and insulin action, has gained attention as potentially beneficial for youth at risk of T1D [111]. It has been associated with improved insulin sensitivity [112,113]. Low magnesium levels are often observed in individuals with poor metabolic health, making supplementation a relevant consideration for at-risk youth.

### 3.3. Impact of Exercise and Physical Activity

#### 3.3.1. Relationship Between Physical Activity, Metabolic Health, and Beta-Cell Preservation

Physical activity is a well-documented factor in improving insulin sensitivity, enhancing glucose metabolism, and supporting metabolic health. Exercise activates pathways in muscle cells that increase glucose uptake, reducing the need for insulin and thereby alleviating some of the metabolic pressure on beta cells [82]. Additionally, regular physical activity has been associated with reduced systemic inflammation, further aiding in beta-cell preservation for adolescents with pre-diabetes. This mechanism is particularly relevant in individuals with impaired glucose tolerance or pre-diabetes, where regular physical activity has been shown to reduce systemic inflammation, which is a contributing factor to beta-cell dysfunction and loss [83]. By mitigating chronic low-grade inflammation, physical activity promotes a more favorable metabolic environment for preserving beta-cell function in adolescents and adults with pre-diabetes.

A specific and practical form of physical activity, walking, provides immediate benefits for postprandial blood glucose regulation. Muscle contractions during walking enhance glucose uptake directly from the bloodstream, bypassing the need for insulin [84]. This effect is particularly pronounced in the post-meal period when blood glucose levels are at their peak. Studies indicate that even light-to-moderate intensity walking for 15–30 min after meals can significantly reduce postprandial glucose spikes, stabilizing glycemic control [114]. This reduction in glucose variability has important implications for individuals at risk of T1D or T2D, as prolonged hyperglycemia is a key driver of beta-cell stress and apoptosis.

Regular post-meal walking also improves insulin sensitivity over time, further supporting glucose homeostasis. Enhanced insulin action reduces the burden on beta cells to secrete insulin, allowing for functional preservation [84]. Additionally, the cardiovascular benefits associated with consistent physical activity, such as improved lipid profiles and weight management, contribute to overall metabolic health and further reduce the risk of beta-cell failure. These findings underscore the role of physical activity not only as a therapeutic intervention for managing blood glucose but also as a preventative measure for beta-cell preservation.

#### 3.3.2. Specific Exercise Recommendations for Adolescents and Teens

For youth at risk of T1D, a combination of aerobic and resistance exercises is recommended [84,115]. Aerobic exercises, such as running, swimming, and cycling, help improve cardiovascular fitness and enhance glucose metabolism, while resistance training builds muscle mass, which further supports glucose uptake and insulin sensitivity [78]. Current recommendations suggest that adolescents should aim for at least 60 min of moderate to vigorous physical activity daily [116]. Integrating age-appropriate, enjoyable activities, such as sports, dance, or active play, can encourage consistency and long-term adherence to physical activity goals [84,115].

### 3.4. Case Studies and Trials

#### Clinical Trials and Observational Studies on Nutrition and Lifestyle Changes

Several clinical trials and observational studies highlight the benefits of nutritional and lifestyle interventions in delaying or preventing the onset of T1D in at-risk populations. For example, the Diabetes Prevention Trial-Type 1 (DPT-1) and TrialNet studies have explored dietary and pharmacological interventions, observing positive outcomes in delaying disease progression among participants with autoantibodies [117]. Observational studies have shown that children with higher adherence to Mediterranean-style diets, characterized by high fruit, vegetable, and whole grain intake, demonstrate reduced markers of inflammation and better glycemic control, underscoring the potential of diet as a preventive measure [87].

In addition, lifestyle interventions that combine diet and physical activity components show promising outcomes in youth populations. For example, the T1D Prediction and Prevention (DIPP) study in Finland indicated that infants and children adhering to dietary guidelines with a lower intake of processed foods exhibited delayed onset of T1D-related autoimmunity [118]. Such findings emphasize the potential of integrated lifestyle interventions for modifying T1D risk factors and support the need for accessible dietary and physical activity education.

## 4. Emerging Interventions

### 4.1. Pharmacological Interventions

#### 4.1.1. Overview of Immune-Modulating Drugs

Immune-modulating drugs are a primary pharmacological strategy to preserve beta-cell function in individuals at high risk of T1D [119]. A comprehensive array of pharmacological interventions for T1D is presented in Table 3. Some of these agents remain under early-phase investigation and have not yet received approval for clinical application. These drugs work by targeting immune cells that mistakenly attack beta cells, slowing down the autoimmune progression toward full-blown diabetes. Teplizumab, a monoclonal antibody that modulates T-cell activity, is the most promising example of an immune-modulating drug [49,51,53] (Table 3). By binding to specific T-cells and preventing their activation, teplizumab can reduce the autoimmune attack on beta cells [120,121,122,123]. Studies such as the TN-10 trial demonstrated that teplizumab delays the onset of T1D by approximately two years in high-risk individuals [50,124] and was the first disease-modifying drug approved to slow disease progression, marking it as a significant step forward in preventive pharmacotherapy for T1D.

Another component, glucagon-like peptide-1 (GLP-1) receptor agonists, traditionally used for managing T2D and obesity, are increasingly being explored for their potential to delay or prevent the onset of T1D. These agents improve glycemic control by enhancing glucose-dependent insulin secretion, suppressing glucagon release, and slowing gastric emptying. Importantly, they exhibit anti-inflammatory properties and promote beta-cell survival and regeneration, which are mechanisms that align with strategies to preserve beta-cell function and delay autoimmune-mediated destruction in T1D [130].

Recent evidence highlights the efficacy of tirzepatide, a dual GLP-1 and glucose-dependent insulinotropic polypeptide (GIP) receptor agonist, in delaying diabetes progression in high-risk populations. In a recent study on adults with pre-diabetes and obesity, treatment with tirzepatide resulted in a 94% reduction in the progression of T2D compared to a placebo over a three-year period [131]. Furthermore, participants achieved an average weight reduction of 22.9%, underscoring the metabolic benefits of this therapy [131]. These findings are significant, as improved metabolic health and reduced systemic inflammation directly contribute to preserving beta-cell function, which is essential in both pre-diabetes and T1D contexts.

While GLP-1 receptor agonists have not yet been extensively studied for preventing T1D, their ability to improve beta-cell survival and modulate the immune response positions them as promising candidates for future clinical trials in at-risk populations, such as individuals with autoantibody positivity or genetic predisposition to T1D. Current research into their anti-inflammatory effects and impact on glucose variability suggests that GLP-1 receptor agonists may complement existing preventive strategies for T1D, particularly when implemented early in the disease course.

#### 4.1.2. Current Trials and Outcomes of Agents Targeting Inflammation and Beta-Cell Preservation

Other agents are under investigation for their potential to delay T1D onset by targeting inflammation and immune responses. Trials with anti-CD20 antibodies (e.g., rituximab) and interleukin-2 (IL-2) therapy have shown some potential in modulating immune responses and preserving beta-cell function, although with varied efficacy in the pediatric population [126,132,133,134]. Abatacept, a drug that interferes with T-cell costimulation, has also shown promise in slowing beta-cell decline [125]. Ongoing trials are evaluating the safety and long-term effects of these drugs, with preliminary data indicating that such treatments may extend beta-cell functionality, potentially delaying T1D onset.

Other pharmacological agents, such as anti-inflammatory drugs and antioxidants, are also being explored [135]. These agents aim to reduce inflammatory cytokine levels around beta cells, thereby protecting them from immune-mediated destruction. Early-stage trials have shown moderate success in reducing inflammation, but more research is needed to determine their effectiveness as standalone therapies or in combination with other immune-modulating agents.

### 4.2. Non-Pharmacological Interventions

Non-pharmacological strategies focus on factors such as stress management and adequate sleep, which are essential for metabolic health [136,137,138,139]. Chronic stress and poor sleep quality elevate cortisol and other stress hormones that increase blood glucose levels, exacerbating beta-cell stress [140,141,142]. Techniques such as mindfulness, cognitive behavioral therapy (CBT), and relaxation exercises are beneficial for at-risk adolescents to help manage stress while establishing regular sleep routines that can support metabolic resilience [85,143,144,145]. Non-pharmacological interventions offer solutions that are quick, easy and require almost nothing to implement. While non-pharmacological interventions cannot completely replace other therapies, they significantly help in preventing the disease.

### 4.3. A Concise Overview of Experimental and Novel Approaches

#### 4.3.1. Advances in Regenerative Medicine and Cell-Based Therapies

Regenerative medicine and cell-based therapies offer innovative possibilities for beta-cell preservation in high-risk individuals [146,147,148,149,150,151,152,153,154,155,156]. However, it is important to note that most of these therapies are currently under clinical trials primarily involving adult T1D patients, with no or very limited data available for adolescent and teen populations. Stem cell-derived beta-cell replacement is an emerging therapeutic avenue where lab-grown beta cells can be transplanted into individuals with compromised beta-cell function [157,158,159,160]. A recent study transplanted chemically induced pluripotent stem cell-derived islets (CiPSCs) beneath the abdominal anterior rectus sheath in an adult T1D patient [153]. This innovative approach aims to restore endogenous insulin production by replacing the destroyed pancreatic β-cells characteristic of T1D. The procedure demonstrated promising outcomes, including improved glycemic control and reduced dependence on exogenous insulin therapy. Notably, the anterior rectus sheath was selected as the transplantation site due to its accessibility and favorable vascularization, which are critical for the survival and function of the transplanted islets. This case represents a significant advancement in T1D treatment, highlighting the potential of CiPSC-derived islets as a viable therapeutic option. However, further research is necessary to assess the long-term efficacy and safety of this approach in a broader patient population. Research on encapsulated beta-cell implants, which protect transplanted cells from immune attack, is ongoing [149,161,162,163,164,165,166]. These implants have shown the potential to restore insulin production and maintain glucose homeostasis in clinical trials in adult T1D patients, though they are still going through experimental phases for adolescents and teens.

Gene therapy is another experimental approach focused on beta-cell preservation [167,168,169,170]. By modifying immune or beta-cell genes, gene therapy aims to reduce immune sensitivity to beta cells or enhance beta-cell resilience [171]. Although this approach is in the early stages of development, gene editing technologies such as CRISPR offer the potential to correct genetic susceptibilities that contribute to beta-cell dysfunction [172].

#### 4.3.2. Role of the Microbiome in Beta-Cell Health and Implications for Pre-Diabetes Interventions

The gut microbiome, which influences immune function and inflammation, has emerged as a potential factor in beta-cell preservation [173]. Alterations in gut microbiota composition have been linked to autoimmune diseases, including T1D [174,175,176,177,178,179]. Preclinical studies indicate that certain gut bacteria may play a role in modulating immune responses and maintaining beta-cell health [180,181,182]. Probiotic and prebiotic interventions that aim to improve gut microbiome balance are currently under investigation as potential pre-diabetes interventions [183,184,185].

Clinical trials are examining whether gut microbiome modulation can reduce T1D risk in at-risk youth by promoting beneficial bacteria that support immune tolerance and reduce autoimmune activity [186]. While research is still in the early stages, the microbiome represents an exciting new development in understanding and could potentially mitigate autoimmune processes that lead to beta-cell destruction.

In summary, beta-cell preservation strategies offer hope for delaying or preventing T1D onset in adolescents and teens. By combining pharmacological interventions, lifestyle modifications, and experimental therapies, researchers aim to slow beta-cell decline and reduce autoimmune attacks in high-risk individuals. Advances in regenerative medicine and an increased understanding of the gut microbiome further broaden the scope of potential interventions. As research progresses, the integration of these strategies will play an essential role in preventive T1D care, ultimately improving the quality of life and long-term outcomes for at-risk youth.

## 5. Social, Emotional, and Psychological Impacts of Pre-Diabetes and T1D Risk

The psychological, social, and emotional implications of pre-diabetes or T1D at-risk diagnosis can be significant for adolescents and their families [187,188]. This stage of life is already marked by considerable emotional and social development, and an added health risk can intensify challenges for both the affected individuals and those around them. Addressing the mental health impacts of pre-diabetes, understanding the role of family and peer dynamics, and providing targeted mental health interventions are essential for supporting these youth. This section delves into these dimensions and highlights the importance of mental healthcare integration in T1D risk management.

### 5.1. Psychological Burden of Screening and Diagnosis

#### 5.1.1. Mental Health Implications of an At-Risk or Pre-Diabetes Diagnosis

An at-risk or pre-diabetes diagnosis in adolescents often brings about a range of emotional reactions, including anxiety, fear, sadness, and confusion [187,188]. Adolescents aware of their potential T1D risk may experience heightened anxiety about the future, particularly regarding the potential development of a chronic, lifelong illness [189,190]. This anxiety can manifest as preoccupation with health, fear of disease progression, and a sense of uncertainty about life goals and physical capabilities.

The mental health burden also extends to families, as parents or guardians of at-risk youth may feel a sense of guilt, worry, and helplessness, fearing that they may not be able to prevent the child’s disease progression [191]. These families often undergo heightened vigilance, closely monitoring the adolescent’s diet, physical activity, and overall lifestyle, which can create family tensions, stress, and parental burnout over time.

#### 5.1.2. Impact of Continuous Glucose Monitoring (CGM) or Self-Monitoring Requirements on Quality of Life

In cases where adolescents are asked to use CGM or conduct self-monitoring as part of their risk management, there can be a significant impact on their quality of life [192]. The routine nature of glucose monitoring can cause feelings of frustration, stress, and self-consciousness, particularly in social settings where self-monitoring or wearing CGM devices may draw unwanted attention [192]. Some adolescents may feel “different” or “isolated” from their peers, leading to social withdrawal, especially in cases where they perceive their health routines as a source of embarrassment [193,194,195].

Moreover, frequent monitoring can lead to hyper-vigilance around blood sugar levels, with adolescents becoming increasingly preoccupied with minor glycemic fluctuations and concerned over the consequences. This heightened focus on health can inadvertently detract from other developmental experiences and contribute to a decline in overall life satisfaction [196].

### 5.2. Family and Peer Dynamics

#### 5.2.1. Influence of Family Dynamics and Peer Relationships on Dietary Adherence and Lifestyle Changes

Family dynamics play a crucial role in the management of pre-diabetes in adolescents [197,198]. The support or lack of support from family members can influence how adolescents adhere to dietary and lifestyle modifications [198]. Supportive families often create environments that prioritize health-conscious meals, encourage regular physical activity, and foster open conversations about feelings related to health risks [199,200]. In such environments, adolescents are more likely to accept and adhere to recommended lifestyle changes, which can significantly improve their quality of life.

Conversely, family conflicts or strained relationships can hinder adherence to lifestyle modifications [201]. For example, families with conflicting dietary preferences or unstructured mealtimes may struggle to implement the recommended dietary changes, creating frustration and resistance in adolescents [202]. Similarly, if family members do not understand the importance of lifestyle modifications, adolescents may feel unsupported in their efforts to adopt healthier habits, potentially increasing feelings of isolation and non-compliance [203].

Peer relationships also influence dietary adherence and lifestyle changes [204]. Adolescents with strong peer support may feel more motivated to adhere to health recommendations if they have friends who encourage or share their health goals. However, peer pressure to engage in unhealthy behaviors, such as consuming sugary or high-fat foods or neglecting physical activity, can challenge an adolescent’s ability to maintain their pre-diabetes management plan [205]. This peer influence can contribute to a heightened risk of non-adherence and potential disease progression.

#### 5.2.2. Importance of Supportive Family Environments and Community Resources

A supportive family environment, combined with access to community resources, can help sustain lifestyle modifications and reduce the mental health burden of a pre-diabetes diagnosis [203,206,207]. Community resources, such as diabetes support groups, nutritional counseling, and physical activity programs, provide adolescents and their families with the knowledge and tools needed for effective disease management [208]. When families participate in community-based programs, they not only reinforce the adolescent’s adherence but also gain social support and a sense of solidarity, which can alleviate feelings of isolation and stress [209].

Community resources that foster social engagement and healthy lifestyle habits allow adolescents to build relationships with others facing similar challenges, creating a sense of belonging and resilience [208]. This sense of connectedness can be instrumental in supporting adolescents’ motivation to follow recommended lifestyle modifications, thus reducing the risk of disease progression [208].

### 5.3. Interventions to Support Mental Health and Coping Skills

#### 5.3.1. Counseling, Group Support, and Cognitive-Behavioral Therapy (CBT) for Youth with Pre-Diabetes and Their Families

Given the significant psychological burden associated with T1D risk, counseling, and therapeutic interventions are crucial for promoting mental well-being in at-risk adolescents (Table 4) [85,143,144,145]. Counseling services that address specific fears, anxieties, and uncertainties about health can empower adolescents to manage their emotions more effectively. Group support programs, which bring together adolescents facing similar challenges, provide a platform for mutual support, encouragement, and the sharing of coping strategies [85,143,144,145]. Group support helps reduce a sense of isolation and provides reassurance that others face similar fears and anxieties [210].

Cognitive-behavioral therapy (CBT) is particularly effective in helping adolescents develop positive coping skills and reframe negative thought patterns associated with their health status (Table 4) [85,143,144,145]. CBT can be tailored to help adolescents manage anxiety related to disease progression, overcome feelings of social isolation, and build resilience to health-related stressors [85,143,144,145]. Moreover, CBT techniques can be extended to family members, helping them manage their own anxieties and developing constructive strategies for supporting the adolescent’s journey.

#### 5.3.2. Integrating Mental Health Services into T1D Prevention Programs

Integrating mental health services into T1D prevention programs provides a holistic approach to pre-T1D management (Table 4) [56,143]. Addressing the mental health needs of adolescents with a high risk of T1D is essential for preventing anxiety, depression, and other mental health issues that can arise in the face of a health challenge [56,143]. Incorporating regular mental health assessments and access to counseling as part of T1D risk management allows for the early detection of emotional difficulties and provides timely interventions to address them [56,143].

Mental health services can also help adolescents build coping skills that are useful in long-term disease management [78]. Stress reduction techniques, such as mindfulness-based stress reduction, relaxation exercises, and guided imagery, can be incorporated into T1D prevention programs to promote emotional stability and reduce health-related anxiety [78]. Additionally, psychoeducational programs that involve families can strengthen family cohesion, enhance communication, and equip family members with strategies to provide effective emotional support [56,143].

## 6. Future Directions and Recommendations

As our understanding of T1D and pre-diabetes in youth continues to advance, a comprehensive strategy integrating screening, preventive care, research, and supportive policies is essential to reduce disease progression and improve the quality of life for at-risk adolescents [78]. Future directions should focus on developing targeted screening initiatives, advancing research in diet and psychosocial care, and advocating for policies that support preventative and educational resources.

### 6.1. Integrating Screening with Preventative Care

#### 6.1.1. Need for Universal or Targeted Screening Programs in Schools or Primary Care

Implementing universal or targeted screening programs in schools and primary care settings can significantly enhance the early detection of T1D risk in children and adolescents [211]. T1D is a chronic autoimmune condition that can affect individuals regardless of their family history [1,2,3,4]. While first-degree relatives of individuals with T1D are approximately 15 times more likely to develop the disease compared to the general population, the majority of new cases—approximately over 80%—occur in individuals without any known family history of the condition [212,213]. This highlights the complex interplay between genetic predisposition and environmental factors in T1D pathogenesis [3], highlighting the need for broader population-based screening strategies to identify at-risk individuals [57]. Universal screening would make it possible to identify at-risk individuals without relying on family history alone, capturing those who may not be aware of their susceptibility [214]. This approach could involve routine blood tests for diabetes-associated autoantibodies and glucose levels during pediatric check-ups or school health programs, allowing healthcare providers to flag at-risk youth early on [17].

However, universal screening has limitations, including the potential for high upfront costs associated with testing entire populations, logistical challenges in ensuring access to screening tools, and the risk of overdiagnosis or false positives, which can lead to unnecessary follow-up procedures and anxiety for patients [77]. These limitations can be addressed through targeted implementation strategies, such as prioritizing high-risk populations like those with a family history of T1D or genetic predispositions [215]. In addition, cost-effective screening technologies such as point-of-care tests can be used to reduce the need for expensive laboratory infrastructure. Standardizing screening protocols and integrating them into existing healthcare systems can further streamline the process and improve efficiency [57]. Emerging strategies, such as combining genetic risk scores with autoantibody testing, can further enhance efficiency [216,217,218]. Despite these challenges, the benefits of universal screening for T1D are substantial, as it enables the early detection of the condition during its pre-symptomatic stages, allowing for timely interventions that can delay disease onset, preserve β-cell function, and improve long-term patient outcomes [63,219]. Early detection through widespread screening can also reduce the risk of life-threatening complications, such as DKA, at initial presentation, thereby decreasing associated healthcare costs over time [63,220]. Moreover, implementing universal screening contributes to more equitable healthcare by identifying at-risk individuals across diverse populations, including those who may lack access to routine medical care [57]. This approach has the potential to not only improve individual outcomes but also advance public health by facilitating preventive measures and personalized management strategies for T1D.

#### 6.1.2. Combining Screening with Family Education and Resources for Early Intervention

Screening alone is not sufficient without the necessary educational and resource-based support to guide families and youth through early interventions [221]. Combining screening efforts with educational resources, such as nutritional counseling and information about lifestyle adjustments, can empower families to adopt preventive measures [222,223,224,225]. Family-centered education programs should include clear guidance on dietary choices, the importance of physical activity, and the benefits of regular health monitoring to mitigate T1D risk factors [197].

This integrated model could extend to providing families with access to online resources, support groups, and trained counselors who can answer questions and offer strategies for managing pre-diabetes risk [222,223,224,225]. Schools, community centers, and pediatric clinics could serve as hubs for disseminating educational materials and connecting families with local health resources, maximizing the impact of early screening initiatives [78].

### 6.2. Policy Implications and Advocacy

#### 6.2.1. Role of Public Health Policies in Supporting Preventive Care, Nutrition, and Mental Health Resources for At-Risk Youth

Public health policies that prioritize preventive care, nutritional resources, and mental health support are essential to reduce the burden of T1D in at-risk youth [78,226]. Policymakers should consider frameworks that promote routine screenings in schools and primary care as part of national health guidelines [226]. Such policies could ensure that screening for diabetes risk is covered for children and adolescents, enabling early identification and timely interventions.

Nutrition-focused policies can support T1D prevention by promoting access to healthy foods in schools, particularly in underserved communities [226]. Programs that provide funding for school meal programs, nutrition education, and access to fruits and vegetables in local markets can help create an environment that encourages healthier dietary choices [78]. Public health policies should also address the mental health needs of at-risk youth by advocating for mental health resources in schools and healthcare facilities, ensuring that adolescents have access to professional support.

#### 6.2.2. Advocacy for Insurance Coverage of Early Screening, Nutritional Counseling, and Psychosocial Support

One of the significant barriers to comprehensive care for pre-diabetes is the lack of insurance coverage for preventive screenings and counseling services [227]. Advocacy efforts are needed to push for insurance policies that cover the costs of screening, nutritional counseling, and mental health support as preventive measures for at-risk youth [228]. The coverage of these services could alleviate the financial burden on families, making it easier for them to access the resources necessary to manage pre-diabetes risk.

Collaborations between healthcare providers, advocacy groups, and policymakers are essential to driving these policy changes. Organizations focused on the prevention of diabetes, such as JDRF and ADA, can play an influential role by presenting data to support the cost-effectiveness of early intervention and the long-term health benefits of preventive measures [19]. Advocacy for broader insurance coverage could support a proactive approach to healthcare, reducing the incidence of T1D and improving the quality of life for youth and families.

## 7. Conclusions

This review highlights the multifaceted challenges and opportunities in addressing pre-diabetes in adolescents and teens at risk of T1D. By synthesizing advancements in screening, nutritional interventions, beta-cell preservation strategies, and psychosocial support, it provides a comprehensive framework for its early detection and management. Unlike previous approaches, this review integrates emerging pharmacological therapies with lifestyle and psychological interventions, emphasizing their synergistic potential that may delay disease onset and improve quality of life.

Future research should prioritize large-scale, longitudinal studies to refine risk stratification models and explore the long-term impacts of these interventions. Additionally, a stronger focus on integrating mental health services and family-centered care into preventive strategies is critical to addressing the emotional burden of a pre-T1D diagnosis. Advocacy for policy reforms that enhance access to screening, nutritional counseling, and mental health resources is essential for reducing health disparities and supporting at-risk populations. As the understanding of T1D pathogenesis evolves, this review highlights the need for a holistic, interdisciplinary approach to prevention and care, aiming to bridge gaps in current practices and improve outcomes for adolescents and their families.

## Figures and Tables

**Table 1:** Screening guidelines for Type 1 Diabetes in adolescent hyperglycemia [17,18,19].

Screening Parameter	Description	Target Population	References
Autoantibody Screening	Detection of islet autoantibodies (such as GAD65, IA-2, and ZnT8) indicating autoimmune activity	Adolescents with a family history of T1D or other autoimmune diseases	[19,21,22]
Genetic Screening	Identification of HLA genotypes associated with increased T1D risk	High-risk individuals, especially those with a first-degree relative with T1D	[16,24,25,26,27,28]
Metabolic Markers	Measurement of C-peptide levels and oral glucose tolerance tests to assess beta-cell function	Adolescents with positive autoantibodies or a genetic predisposition	[29,30,31,32,33,34,35]
Continuous Glucose Monitoring (CGM)	The use of CGM devices to detect dysglycemia in at-risk adolescents	Adolescents with positive autoantibodies or early signs of glucose intolerance	[36,37,38,39,40,41,42,43]

**Table 2:** Nutritional and lifestyle interventions for adolescents with pre-diabetes.

Intervention	Description	Expected Outcome	References
Low Glycemic Index Diet	Emphasis on consuming foods with a low glycemic index to stabilize blood sugar levels	Improved glycemic control and reduced insulin resistance	[80]
Mediterranean Diet	A diet rich in fruits, vegetables, whole grains, and healthy fats	Reduction in inflammation and improvement in insulin sensitivity	[81]
Physical Activity	Regular aerobic and resistance training exercises	Enhanced glucose uptake and improved cardiovascular health	[82,83,84]
Behavioral Counseling	Structured programs focusing on lifestyle modification	Increased adherence to healthy behaviors and weight management	[85]

**Table 3:** Emerging pharmacological interventions for pre-diabetes and Type 1 Diabetes (T1D).

Drug Class	Example Agent	Mechanism of Action	Clinical Trials. gov Number	Current Research Status	Expected Outcomes	Side Effects/Considerations	References
Immune Modulators	Teplizumab	Anti-CD3 monoclonal antibody modulating T-cell activity to reduce autoimmune beta-cell attack	NCT01030861	Phase III trials; shown to delay T1D onset	Delay in progression to clinical T1D; preservation of beta-cell function	Possible immunosuppression; monitoring required	[39]
	Abatacept	Interferes with T-cell costimulation to reduce immune response against beta cells	NCT01773707	Early clinical trials	Slower beta-cell decline, and potential delay in T1D onset	Mild-to-moderate injection site reactions	[125]
	Anti-CD20 (e.g., rituximab)	Depletes B-cells to modulate autoimmune activity	NCT00279305	Phase II trials; varied efficacy	Reduction in autoimmune activity; partial beta-cell preservation	Risk of infection due to B-cell depletion	[50,53]
Anti-Inflammatory Agents	Interleukin-2 (IL-2)	Low-dose IL-2 therapy aimed at enhancing regulatory T-cell function	NCT01862120	Ongoing clinical studies	Improved immune tolerance; reduced beta-cell stress	Requires dosage optimization; risk of fever	[126]
Beta-Cell Modulators	Verapamil	Calcium channel blocker potentially inhibiting beta-cell apoptosis	NCT02372253	Phase II trials; promising in T1D	Beta-cell survival enhancement; improved glucose control	Dizziness; low blood pressure	[127]
	GLP-1 receptor agonists	Enhancing beta-cell proliferation and insulin secretion in response to glucose	NCT02846545	Phase III for T2D, exploratory for T1D	Increased beta-cell function and insulin sensitivity	Nausea; potential risk of pancreatitis	[128]
Other Novel Agents	Treg-enhancing agents	Agents aiming to boost regulatory T-cell numbers and function	NCT03182426	Preclinical and early clinical trials	Enhanced immune tolerance; delayed beta-cell decline	Long-term safety under investigation	[129]

**Table 4:** Psychosocial and mental health interventions for adolescents at risk of T1D.

Intervention	Description	Expected Outcome	References
Cognitive Behavioral Therapy (CBT)	Techniques to manage anxiety and stress related to health monitoring and disease risk	Reduced anxiety levels and improved coping mechanisms	[85,143,144,145]
Family Therapy	Counseling sessions involving family members to improve communication and support	Enhanced family cohesion and better adherence to lifestyle interventions	[56]
Peer Support Groups	Group sessions with peers facing similar health challenges	Increased emotional support and shared coping strategies	[210]
Stress Management Programs	Programs teaching stress reduction techniques such as mindfulness and relaxation exercises	Lowered stress levels and improved overall well-being	[56,143]

## Data Availability

Not applicable.
